# Safety reporting on implantation of autologous adipose tissue-derived stem cells with platelet-rich plasma into human articular joints

**DOI:** 10.1186/1471-2474-14-337

**Published:** 2013-12-01

**Authors:** Jaewoo Pak, Jae-Jin Chang, Jung Hun Lee, Sang Hee Lee

**Affiliations:** 1 Stems Medical Clinic, Fourth Floor, 32-3 Chungdam-dong, Gangnam-gu, Seoul 135-950, Republic of Korea; 2 Oriental Bio Group, Seongnam, Republic of Korea; 3 National Leading Research Laboratory, Department of Biological Sciences, Myongji University, 116 Myongjiro, Yongin, Gyeonggido 449-728, Republic of Korea

**Keywords:** Mesenchymal stem cell, Adipose tissue-derived stem cells, Platelet-rich plasma, Complications, Safety, Orthopedics

## Abstract

**Background:**

Adipose tissue-derived stem cells (ADSCs), a type of mesenchymal stem cells (MSCs), have great potential as therapeutic agents in regenerative medicine. Numerous animal studies have documented the multipotency of ADSCs, showing their capabilities to differentiate into tissues such as muscle, bone, cartilage, and tendon. However, the safety of autologous ADSC injections into human joints is only beginning to be understood and the data are lacking.

**Methods:**

Between 2009 and 2010, 91 patients were treated with autologous ADSCs with platelet-rich plasma (PRP) for various orthopedic conditions. Stem cells in the form of stromal vascular fraction (SVF) were injected with PRP into various joints (*n* = 100). All patients were followed for symptom improvement with visual analog score (VAS) at one month and three months. Approximately one third of the patients were followed up with third month magnetic resonance imaging (MRI) of the injected sites. All patients were followed up by telephone questionnaires every six months for up to 30 months.

**Results:**

The mean follow-up time for all patients was 26.62 ± 0.32 months. The follow-up time for patients who were treated in 2009 and early 2010 was close to three years. The relative mean VAS of patients at the end of one month follow-up was 6.55 ± 0.32, and at the end of three months follow-up was 4.43 ± 0.41. Post-procedure MRIs performed on one third of the patients at three months failed to demonstrate any tumor formation at the implant sites. Further, no tumor formation was reported in telephone long-term follow-ups. However, swelling of injected joints was common and was thought to be associated with death of stem cells. Also, tenosinovitis and tendonitis in elderly patients, all of which were either self-limited or were remedied with simple therapeutic measures, were common as well.

**Conclusions:**

Using both MRI tracking and telephone follow ups in 100 joints in 91 patients treated, no neoplastic complications were detected at any ADSC implantation sites. Based on our longitudinal cohort, the autologous and uncultured ADSCs/PRP therapy in the form of SVF could be considered to be safe when used as percutaneous local injections.

## Background

Mesenchymal stem cells (MSCs), including ADSCs, have great potentials as a future therapeutic agent in the field of regenerative medicine. This has created much *in vitro*[1-3], *in vivo*[4-14], and clinical [15-26] experimentations on MSCs. MSCs can be readily extracted from bone marrow of hip and spine, and adipose tissue of abdomen, thigh and hips [27,28]. These extracted MSCs have potentials to differentiate into bone, cartilage, tendon, muscle, and other tissues [15-17]. Such capabilities of MSCs to differentiate into bones and cartilage have been documented by numerous animal studies [9-14]. Also, there have been few reports of successful regeneration of bone and cartilage in humans by using various MSCs [16-26], particularly ADSCs [19-23].

\The platelets contain critical growth factors and mediators of tissue repair pathways. Activation of platelets with calcium chloride has been shown to induce immediate platelet growth factor release *in vitro*[29]. The platelet rich plasma (PRP) obtained from autologous blood contains a high concentration of stored autologous growth factors. By exposing PRP to calcium chloride, degranulation of platelet is induced. PRP has been successfully used as a cell culture additive to facilitate growth and differentiation of autologous MSCs [30-33].

However, the safety data for using the autologous MSC therapy is lacking. Although Centeno *et al*. has reported safety and complication rate of using autologous bone marrow derived stem cells in orthopedic applications [34], there has not been any safety report on using ADSCs for human orthopedic applications. One of the safety issues that need to be addressed is the potential of these stem cells becoming neoplasms. This concern was brought on by reports of chromosomal abnormalities in MSCs (not including ADSCs) that have been cultured *in vitro*[35-38]. However, when these cells are cultured less than 60 days *in vitro*, they pose no detectable risk of cell mutation or tumor formation, as previously reported [39]. Further, autologous MSCs that have been used for cartilage repair in humans also indicate that they form no tumors during long-term follow-up [26]. The few early human clinical trials using MSCs completed thus far support the conclusion that MSCs are safe as therapeutic agents [30,34,40]. However, these MSCs were not including ADSCs.

The purpose of this study was to examine the safety profile of ADSCs along with PRP for human orthopedic applications into articular joints.

## Methods

In 2009, Korean Food and Drug Administration (KFDA) has approved medical uses of autologous ADSCs with minimal manipulation [41]. From November 2009 until December 2010, 91 patients with 100 articular joints were treated with autologous ADSCs in the form of stromal vascular fraction (SVF) along with autologous PRP. The mixture of ADSCs and PRP were percutaneously injected into knees, hips, low backs, and ankles. After the completion of the treatment, all patients were followed for three months. At the third month, some of the patients were also followed up with high field MRI of the implant sites. Afterwards, all patients were followed up with telephone questionnaires every six months: at 6, 12, 18, 24, and 30 months.

This study was in compliance with the Declaration of Helsinki and regulation guidelines of KFDA. The written informed consent for participation in the study was obtained from participants. According to Korean law (Rules and Regulations of the Korean Food and Drug Administration), questionnaire and register based studies do not need approval by ethical and scientific committees, and do not require informed consent [41]. All data was de-identified and analyzed anonymously.

### Inclusion and exclusion criteria and outcome endpoints of patients

For all patients, aspirin, NSAIDS, or any other form of anticoagulant therapy were stopped at least seven days prior to liposuction and resumed one week after. Aspirin were continued to be withheld until the last day of PRP injection.

The inclusion criteria, exclusion criteria, and outcome endpoints were listed as follows: Inclusion criteria: (i) age 18 and older; (ii) chronic or degenerative joint disease causing significant functional disability and/or pain; (iii) the failure of conservative treatments; and (iv) an unwillingness to proceed with surgical intervention.

Exclusion criteria: (i) active inflammatory or connective tissue disease thought to impact pain condition (i.e., lupus, rheumatoid arthritis, and fibromyalgia); (ii) active non-corrected endocrine disorder that might impact pain condition (i.e., hypothyroidism and diabetes); (iii) active neurologic disorder that might impact pain condition (i.e., peripheral neuropathy and multiple sclerosis); (iv) pulmonary and cardiac disease uncontrolled with medication usage; (v) history of active neoplasm within the past five years; (vi) blood disorders documented by abnormal complete blood count (CBC) within three months including severe anemia, thrombocytopenia, leukocytosis and/or leukopenenia; and (vii) medical conditions precluding the injection procedures.

Outcome endpoints (pain score, imaging, and telephone questionnaires): (i) pre-treatment visual analog scale (VAS); (ii) VAS at one month after the procedure; (iii) VAS at three months after the procedure; (iv) MRI imaging within three months before the ADSC injection procedure; and (v) MRI imaging at three months after the procedure; (vi) telephone questionnaires every six months: at 6, 12, 18, 24, and 30 months.

### Liposuction procedure

Patients were restricted from taking corticosteroids, aspirin, non-steroidal anti-inflammatory drugs (NSAIDs), and oriental herb medications for minimum one week prior to the liposuction.

For the liposuction procedure, the patients were sedated with propofol 2 mg IV push and 20–30 mg/h rate of continuous infusion.

Using tumescent solution (40 mL of lidocaine [20 g/L] with 20 mL of Marcaine [5 g/L], 500 mL normal saline, and 0.5 mL of epinephrine 1:1000), the lower abdomen area was anesthetized. Next, lipoaspirates were extracted and separated by gravity. The resulting 100 mL of adipose tissue with the tumescent solution were then centrifuged at 1000 *g* for 5 minutes. The end result was approximately 40 mL of packed adipose tissue, fibrous tissue, red blood cells and a small number of nucleated cells. An equal volume of digestive enzyme (0.07% type 1 collagenase; Adilase, Worthington, Lakewood, NJ, USA) was then mixed with the centrifuged lipoaspirates at a ratio of 1:1. The mixture was then digested for 30 min at 37°C while rotating. After the digestion, the lipoaspirates were centrifuged at 100 *g* for 3 minutes to separate the lipoaspirate and the enzyme. The left-over enzyme was then removed.

Using 5% dextrose in lactated Ringer’s solution (D5LR), the lipoaspirates were washed three times to remove the collagenase. After each washing, the lipoaspirates were centrifuged at 100 *g*. After the last centrifuge, approximately 10 mL of ADSCs-containing SVF were obtained [28].

### PRP preparation

While preparing the ADSCs, 30 mL of autologous blood was drawn with 2.5 mL anticoagulant citrate dextrose solution (0.8% citric acid, 0.22% sodium citrate, and 0.223% dextrose; Baxter Healthcare Corp., Marion, NC, USA). This was centrifuged at 200 *g* for 10 minutes. The supernatant was drawn and spun at 1000 *g* for 5 minutes. The supernatant was drawn and discarded. The resulting 2 mL buffy coat (PRP) was mixed with 10 mL of ADSCs-containing SVF. To this mixture, 0.5% (w/v) hyaluronic acid (1 mL; Huons, Chungbuk, Korea) was added as a scaffold. This mixture was again mixed with CaCl_2_ at a ratio of 10:2 (PRP to CaCl_2_) for activation of the platelets. This autologous PRP mixture was freshly prepared on the same day of liposuction. Afterwards, when patient visited for weekly PRP injection, it was freshly prepared each time.

### ADSCs injection with PRP

On the day of liposuction, ADSCs and PRP were prepared within the same surgical procedure. In order to inject ADSCs with PRP, the patients’ joints were prepared by sterile technique and were anesthetized with 2% lidocaine. Then, the mixture of ADSCs, PRP, hyaluronic acid, and CaCl_2_ was injected into the joint under the ultrasound guidance at an aseptic room.

The patients were then instructed to remain still for 30 minutes to allow for cell attachment. As they were discharged to home, the patients were instructed to maintain activity as tolerated.

The patients returned for four additional autologous PRP injections that were freshly prepared with CaCl_2_ each week over one month period.

### The follow-up disease surveillance questionnaires

All patients were followed up with telephone questionnaires every six months: at 6, 12, 18, 24, and 30 months. Each time, patients were asked the following questions: (i) did you experience any complications (i.e., infection, illness, etc.) you believe may be due to the procedure? If yes or maybe, please explain; and (ii) have you been diagnosed with any form of cancer since the procedure? If yes, please explain.

### Statistics

Statistical analysis was performed using SPSS software version 20.0 (SPSS Inc., New York, USA). There were three groups (pre-treatment, one month after treatment, and three months after treatment) and Levene’s test results revealed that the variance of the dependent variable (relative VAS) was not equal across groups. Therefore, Kruskall-Wallis test was conducted to compare the means of three groups. To detect significant differences among groups, post hoc testing was performed by Mann–Whitney U tests. A *p*-value below 0.05 was considered statistically significant.

## Results

### Patient demographics and areas treated

From November 18, 2009 until December 17, 2010, a total of 100 joints were treated in 91 patients. As of October 2012, the mean follow-up time for all patients was 26.62 ± 0.32 months. 100 procedure follow-up contacts occurred at 12 months or more, 78 contacts at 24 months or more, and 17 contacts at 30 months (Table 1). The first patient was treated on November 18, 2009. Thus, the follow-up time for patients who were treated in 2009 and early 2010 was close to three years.

**Table 1:** Number of follow-up contacts at each end-point in the six age ranges

**Age range (years)**	**Number of follow-up contacts**				
	**1 mo.**	**3 mo.**	**12 mo.**	**24 mo.**	**30 mo.**
18 ~ 29	9	9	9	7	2
30 ~ 39	15	15	15	12	-
40 ~ 49	23	23	23	15	3
50 ~ 59	19	19	19	15	4
60 ~ 69	21	21	21	18	4
70 ~ 80	13	13	13	11	4

-: no contact.

There were a total of four patients in 2009. They were all female and their age was 61, 57, 71, and 77, respectively. Of these four, the first patient received two injections of ADSCs into the knee: the first dose was injected on November 18, 2009, and the second dose was injected on April 28, 2010. The first dose of the first patient did not include PRP. Thus, the procedure was repeated on April 28, 2010 with PRP and ADSCs. Of the total of 91 patients, three patients received two treatments of identical joints and six patients received two treatments at different joints. Nine patients underwent two procedures. Of the nine, three patients received ADSCs on the same knee joints and the other six patients received ADSCs on the other knees. The second procedure of all nine patients occurred with 3 ~ 4 months after the first. Of these nine patients, only one patient did not obtain MRI after the first and second doses of ADSCs. All other eight patients obtained MRI after three months from each first and second ADSCs/ PRP treatment. Thus, the total number of joints treated (*n* = 100) is higher than the number of patients (*n* = 91) treated. The mean age was 51.23 ± 1.50, with the range being 18–78 years; 45 patients were male and 46 female. The age of patients was relatively evenly distributed (Table 1). Nine patients underwent more than one procedure. In all, the patients underwent 74 knee procedures (distributed with 67.6% in the age range of 40 ~ 69), 22 hip procedures (distributed with 81.8% in the age range of 18 ~ 49), two low back procedures, and two ankle procedures (Table 2). Of the 22 hip procedures, 15 were avascular necrosis and seven were hip osteoarthritis. All 74 knees and two ankles had diagnosis of osteoarthritis while the two low back procedures had spinal disc herniation.

**Table 2:** Frequency of sites treated in all patients separated into the six age ranges

**Age range (years)**	**Number of sites treated**			
	**Knee**	**Hip**	**Low back**	**Ankle**
18 ~ 29	4	5	-	-
30 ~ 39	8	6	-	1
40 ~ 49	14	7	1	1
50 ~ 59	17	2*	-	-
60 ~ 69	19	2*	-	-
70 ~ 80	12	-	1	-

-: no site treated; *: two sites including hip bone and hip cartilage.

Pre-procedure MRIs were carried out on 86 patients. Among these, 27 patients also underwent post-procedure MRIs. Due to financial reasons, 59 patients refused to undergo post-procedure MRIs. Mean time for last MRI follow-up since the procedure was 3.11 ± 0.12 months. MRIs (as read by both examiners and physicians) were negative for any evidence of tumor formation at the implantation site.

### Pain measurements

To assess the symptom improvement of the treatment, VAS was analyzed. For all patients, VAS was marked at 10 before treatments. The patients were followed up consequently with VAS at one month and at three months after the initial injection. Thus, the VAS results are relative to the VAS marked before the initial ADSCs-containing SVF injections.

**Figure 1:**
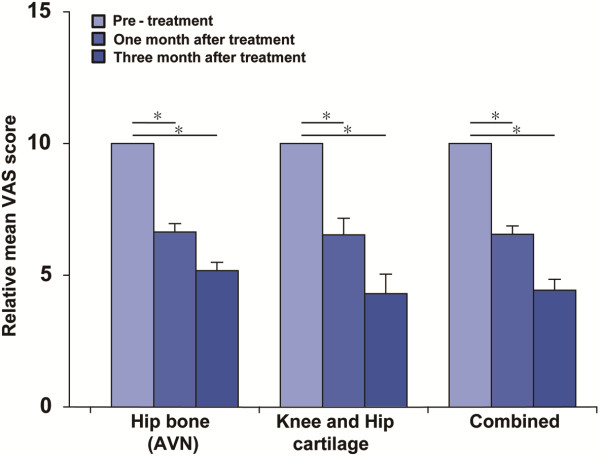
**Pain measurements of patients.** VAS and AVN are visual analog scale and avascular necrosis, respectively. Combined relative mean VAS scores (right three bars) are those of 96 joints (hip bone [left bars]: 15 joints; knee and hip cartilage [middle bars]: 81 joints). Error bars indicate standard errors. * = *p* < 0.05.

The relative mean VAS of 96 joints at the end of one month follow-up was 6.55 ± 0.32, and at the end of three months follow-up was 4.43 ± 0.41 (Figure 1). The relative mean VAS of cartilage repair of 81 joints (74 knees and seven hips; excluding two ankles and two low backs) at the end of one month follow-up was 6.53 ± 0.63 and at the end of three months follow-up was 4.30 ± 0.74 (Figure 1). The lowest values of the relative mean VAS were in the age range group of 70 ~ 80 (Table 3). The relative mean VAS of bone regeneration (*n* = 15) in hips of AVN patients at end of one month follow-up was 6.64 ± 0.32 and at end of three months follow-up was 5.17 ± 0.32 (Figure 1). Two low back and ankle patients reported minimal clinical improvement and did not obtain post procedure MRIs.

**Table 3:** Pain measurements of patients separated into the six age ranges

**Age range (years)**	**Relative mean VAS score**								
	**Hip bone (AVN)**			**Knee and Hip cartilage**			**Combined**		
	**Pre**	**1 mo.**	**3 mo.**	**Pre**	**1 mo.**	**3 mo.**	**Pre**	**1 mo.**	**3 mo.**
18 ~ 29	10	8.50 ± 0.33	-	10	5.80 ± 1.40	3.00 ± 0.62	10	6.57 ± 1.53	3.00 ± 1.38
30 ~ 39	10	5.20 ± 0.83	3.40 ± 0.54	10	7.71 ± 0.75	5.86 ± 0.89	10	6.67 ± 1.30	4.83 ± 1.64
40 ~ 49	10	6.33 ± 0.25	6.33 ± 0.23	10	6.38 ± 1.67	4.45 ± 1.59	10	6.28 ± 1.71	4.89 ± 1.97
50 ~ 59	10	10*	6*	10	6.81 ± 1.10	4.21 ± 1.27	10	7.20 ± 1.16	4.31 ± 1.99
60 ~ 69	10	9*	9*	10	6.75 ± 1.85	4.97 ± 1.60	10	6.75 ± 1.83	5.21 ± 1.92
70 ~ 80	-	-	-	10	5.00 ± 0.69	2.44 ± 0.62	10	5.00 ± 0.97	2.44 ± 1.93

Pre: pre-treatment; 1 mo.: one month after treatment; 3 mo.: three months after treatment; -: not determined; *: VAS score of one patient.

### Complications reporting

The complications of the concern can be summarized as follows: (i) *pain and swelling*: 37% of 100 joints treated joints reported development of pain and swelling after one day after the ADSCs/PRP injection. Pain swelling improved with cold compressions and routine oral NSAIDs prescriptions given on the first day of discharge. These complications were detected only in the knee joints treated. Among these patients, 51% were older than 50; (ii) *infection*: 0%; (iii) *neurologic*: 1% (a hemorrhagic stroke approximately two weeks after the procedure); (iv) *tumor*: 0%: (v) *tendonitis/tenosynovitis*: 22%. Among these patients, 68% were older than 60 and started to occur after 6 ~ 8 weeks of the procedure; and (vi) *skin*: 1% (a localized rash around the injection site after 1 ~ 2 days of the procedure) (Table 4).

**Table 4:** Frequency of complications reported in the six age ranges

**Age range (years)**	**Percentage of treated joints**					
	**Pain and swelling with improvement**	**Infection**	**Neurologic**	**Tumor**	**Tendonitis/ Tenosinovitis**	**Skin**
18 ~ 29	3	-	-	-	-	-
30 ~ 39	5	-	-	-	-	-
40 ~ 49	10	-	-	-	2	-
50 ~ 59	10	-	-	-	5	-
60 ~ 69	4	-	1	-	10	1
70 ~ 80	5	-	-	-	5	-

-: no complication.

## Discussion

Our results show that autologous and uncultured ADSCs/PRP therapy in the form of SVF could be considered to be safe when used as percutaneous local injections.

ADSCs were extracted using collagenases, which were later removed by washing the ADSCs with D5LR solution three times. After all three washings, the total amount of collagenase contained in the ADSCs was undetectable (data not shown). Therefore, it would be very doubtful if such undetectable amount of collagenase would have caused swelling and pain of the joints. However, it can be assumed that some of the cells injected into the joints, including red blood cells and ADSCs, would not have survived, as previously reported [42]. Probably such non-viable cells surmount an inflammatory reaction, causing pain and swelling. A recent report supports this notion [43]. Such pain and swelling subside gradually with oral intakes of NSAIDs and cold compression. The frequency of these complications was more than that of other report using culture-expanded bone marrow-derived MSCs [34].

After 6–8 weeks of ADSCs and PRP injection of knee joints, some elderly patients complained of knee pain secondary to tenosinovitis and tendonitis. These symptoms usually occurred in elderly patients over the age of 60 (15 of 22 patients), as previously described [44]. These symptoms improved with NSAIDs and resolved with trigger point injections with triamcinolone at third or fourth months. The exact cause of these symptoms is not clear. It can be postulated that knee posture may be the cause. Cartilage regeneration, decreased mobility during the procedure, or increased mobility after the procedure may cause the positioning of the knee joint to be changed, resulting the tendons and ligament to strain, as previously reported [45].

One patient experienced a localized rash around the injected site of the knee. Another patient experienced a hemorrhagic stroke. Although the percentage of these complications is greater than 1% of the total patient population of this group, these two are more of co-incidences. The likely causative agent in case of the localized rash would be the synthetic hyaluronic acid that was injected as a scaffold material. As for the hemorrhagic stroke, no other patient experienced such symptoms in the other patient group treated after the year 2010. Further, in the case of hemorrhagic stroke, it is difficult to assume that any form of cells injected locally with PRP were re-absorbed or transferred to blood stream, causing hemorrhagic stroke. In addition, recent research by Horie *et al*. demonstrated that MSCs implanted in a joint remain localized to the transplant site [46].

According to the results of the analysis of pain scores and telephone questionnaires from 100 joints, there has been no report of tumor formation at the implant sites. Further, there was no evidence of tumor formation in 27 joints that were imaged by MRI. This study is the first report on ADSCs safety in clinical applications. While it is possible that tumors may still form at some time beyond the average follow-up period presented in our data, this possibility decreases at an exponential rate. MSCs replicate every 2–4 days *in vitro* expansion [35-37]. Thus, if a tumor were to form at the implanted site, it might have been discernible within few months by a high field MRI.

Patients’ VAS after the ADSCs/PRP treatment improved 50-60%, and this was statistically significant (Figure 1). The pain improvement can be attributed to the probable regeneration of the damaged tissues that have been documented by MRI in some of the patients. However, no patient reported 100% resolution of the pain. Many possibilities exist for the explanation of incomplete resolution of the pain. One of the reasons would be the fact that the extent of the regeneration was achieved only partially. Another possible rationale for the incomplete resolution of the pain is probably the fact that the osteoarthritis and AVN are joint disorders as a whole, not just degeneration of only cartilage and bone, respectively.

The two low back and the two ankle patients reported minimal clinical improvement. Many reasons exist for possible rationale of the minimal pain improvement. Also, quantity and quality of MSCs are important. Another important factor is properly targeting the site for injection [46], especially if the target site has limited space. With regards to low backs, uncertainty exists if ADSCs were correctly and properly placed in the disc. As for the ankle, the quantity and quality of ADSCs injected in the limited joint space could be questioned.

Overall, this study with 100 joint injections of ADSCs, in the form of SVF, with PRP shows that ADSCs/PRP treatment is safe and provides long-term pain improvement. However, this study only shows a glimpse of the possibility of using ADSCs in the field of regenerative medicine. All patients were offered the third month post-procedure MRIs, but some patients refused to undergo post-procedure MRIs due to symptom improvement and/or financial reasons. Therefore, the longer-term (more than 3 months) follow-ups were conducted based on telephone questionnaires. The measurement of PRP concentration and the assessment of viable ADSCs’ quantity and quality are necessary. Also, randomized, double blind, and placebo-controlled studies are needed to confirm the efficacy of ADSCs in various joint diseases.

## Conclusions

This retrospective cohort study demonstrated no evidence of neoplastic complications in any implant sites in 91 patients with 100 joints, some of whom were monitored with high field MRI tracking and via general surveillance. In summary, based on this longitudinal cohort, autologous and uncultured ADSCs/PRP therapy in the form of SVF can be considered to be safe when used as percutaneous local injections.

## Abbreviations

ADSC: Adipose tissue-derived stem cell; MSC: Mesenchymal stem cell; PRP: Platelet-rich plasma; SVF: Stromal vascular fraction; MRI: Magnetic resonance imaging.

## Competing interests

The authors declare that they have no competing interests.

## Authors’ contributions

JP and SHL contributed to the conception and design of the study, data acquisition, analysis and interpretation. JJC and JHL contributed to the data acquisition, analysis and interpretation. JP and SHL performed the literature review and drafted the manuscript. All of the authors performed a critical review of the manuscript and approved the final version of the manuscript submitted for publication.

## Pre-publication history

The pre-publication history for this paper can be accessed here:

http://www.biomedcentral.com/1471-2474/14/337/prepub
